# Chemo-Preventive Effect of Vegetables and Fruits Consumption on the COVID-19 Pandemic

**Published:** 2021-03-25

**Authors:** Clement G Yedjou, Richard A Alo, Jinwei Liu, Juliet Enow, Pierre Ngnepiepa, Richard Long, Lekan Latinwo, Paul B Tchounwou

**Affiliations:** 1 Department of Biological Sciences, College of Science and Technology, Florida Agricultural and Mechanical University, 1610 S. Martin Luther King Blvd, Tallahassee, FL 32307, USA; 2 Department of Computer Science, College of Science and Technology, Florida Agricultural and Mechanical University, 1610 S. Martin Luther King Blvd, Tallahassee, FL 32307, USA; 3 Department of Health Policy and Administration. School of Public Health, Jackson State University, 350 W. Woodrow Wilson Drive, Jackson, MS 39213, USA; 4 Department of Mathematics, College of Science and Technology, Florida Agricultural and Mechanical University, 1610 S. Martin Luther King Blvd, Tallahassee, FL 32307, USA; 5 Department of Biology, College of Science, Engineering and Technology, Jackson State University, 1400 Lynch Street, Box 18750, Jackson, MS 39217, USA

**Keywords:** Chemo preventive agents, COVID-19, Death rate, Fruits, Incidence rate, Vegetables

## Abstract

Coronavirus disease 2019 (COVID-19) is a new disease caused by the novel severe acute respiratory syndrome coronavirus 2 (SARS-CoV-2). It is a global pandemic that has claimed the death of 1,536,957 human beings worldwide including 287,842 deaths in the United States as of December 3, 2020. It has become a major threat to the medical community and the entire healthcare system in every part of the world. Recently, the Food and Drug Administration (FDA) has approved the emergency use of Pfizer and Moderna COVID-19 vaccine on December 12, 2020. However, there are concern about the new COVID-19 vaccine safety, efficacy, and immunity after the vaccination. In addition, both coronavirus and COVID-19 vaccine are new at this point and there is no scientific evidence to know whether people who are vaccinated can still carry the COVID 19 pathogens and pass them along to others. Therefore, many people all over the world have an increased interest in consuming more VF for the purpose of maintaining their health and boosting their immune system. Identifying novel antiviral agents for COVID-19 is of critical importance, and VF is an excellent source for drug discovery and therapeutic development. The objective of this study is to test the hypothesis that a high intake of vegetables and/or fruits prevents COVID-19 incidence and reduces the mortality rate. To achieve this objective, we collected the diet data of COVID-19 from Kaggle (https://www.kaggle.com/mariaren/covid19-healthy-diet-dataset), and used a machine-learning algorithm to examine the effects of different food types on COVID-19 incidences and deaths. Specifically, we used the feature selection method to identify the factors (e.g., diet-related factors) that contribute to COVID-19 morbidity and mortality. Data generated from the study demonstrated that VF intake can help to combat the SARS-CoV-2. Taken together, VF may be potential chemopreventive agents for COVID-19 due to their antiviral properties and their ability to boost the human body immune system.

## Introduction

The coronavirus disease 2019 (COVID-19) is a new disease caused by Severe Acute Respiratory Syndrome Coronavirus-2 (SARS-CoV-2) [1]. Transmission of SARS-CoV-2 (COVID-19) from human-to-human has been reported in healthcare, family, and community. COVID-19 is usually transmitted through the respiratory tract via droplets or fomites and to some extent via aerosols when an infected person coughs, speaks, or sneezes [2–4]. A healthy person can also be infected with the virus by touching contaminated surfaces and then touching the nose, eye, or mouth without washing the hands [5,6].

This disease has generated a serious health crisis that has affected many lives and killed thousands of people worldwide. The numbers of COVID-19 cases and deaths continue to gradually increase in the United States and around the world. Some COVID-19 patients develop severe illness such as decreased percentages of basophils, eosinophils and monocytes, increased percentages of neutrophils to lymphocytes, respiratory failure, acute respiratory distress syndrome, multiple organ failure, and even death [7–9]. Until November 2020, there was no vaccine or cure for COVID-19 but there were numerous ongoing efforts for the development of a vaccine [10,11]. Interestingly the Drug and Food Administration (FDA) recently approved a COVID 19 vaccine developed by Pfizer and Moderna and it is now available as key to prevent COVID 19 pandemic and eradicate this disease. In addition to the COVID 19 vaccine, the United States federal government continues to take a strict approach to fighting against the virus by encouraging each state to implement social distancing, hand washing, shelter-in-place, environmental hygiene, and wearing face masks. In addition to strict approaches taken by the U.S. government, people are seeking preventive ways to protect themselves from the COVID-19 virus. One such preventive way which is well-documented in the literature and often in the news is the consumption of Vegetables and Fruits (VF). The beneficial health effects of VF consumption have been attributed to their high contents of micronutrients, dietary fibers, and bioactive components [12–15].

A large body of scientific studies demonstrates that healthy eating patterns associated with regular exercise can help people have a healthy life, maintain good health, and reduce the risk of many diseases (cancer, diabetes, cardiovascular disease, etc.) throughout all stages of the lifespan. However, many countries, in particular most developed nations, consume low levels of VF. For example, the levels of VF consumption by the majority of adults in the United States is below the recommendations according to the Dietary Guidelines [16–18]. According to the 2015–2020 Dietary Guidelines for Americans, an American adult should consume 2 to 3 cups of vegetables per day and 1.5 to 2 cups of fruits per day depending of the sex and age as part of an overall heathy diet [19]. Despite the positive health benefits of eating VF, many Americans continue to eat insufficient amounts. VF is important sources of many nutrients such as dietary fiber, folic acid, micronutrients, vitamins, and minerals. Several VF such as *Allium sativum* (garlic) [20,21], ascorbic acid (vitamin C) [22,23], curcumin [24–26], green tea [27,28], and *Nigella sativa* (black seed) [29–32] have shown antiviral properties for the prevention and/or treatment of coronavirus 19 disease. Traditionally, these VF have been used to improve human health and prevent and/or treat many diseases. Experimental trials have documented their antioxidant, anti-fungal, anti-microbial, anti-cancer, and anti-inflammatory activities [33–40].

Since the start of the COVID-19 outbreak in many countries around the world, scientists have made significant progress to identify VF that may prevent or cure SARS-CoV-2. Therefore, it is logical to explore VF for the prevention of the virus that causes COVID-19. The aim of this study was to use machine learning to analyze VF intake data from populations in both developed and developing nations and test the hypothesis that high VF intakes may lower the incidence and mortality rates of COVID-19.

## Approaches

### Dataset

In this section, we describe our dataset. We collected diet data of COVID-19 from Kaggle (https://www.kaggle.com/mariaren/covid19-healthy-diet-dataset) for our experimental analysis. The dataset includes relevant information on different types of food, world population obesity, and undernourished rate, and global COVID-19 cases from around the world. The data for different food groups including quantities, nutritional values, obesity, and undernourished percentages were obtained from the United Nations Food and Agriculture Organization (UNFAO) website. The data for COVID-19 confirmed cases, deaths, recovered cases, and active cases were obtained from the Johns Hopkins Center for Systems Science and Engineering (CSSE) website. In this paper, we focus on analyzing the effects of some food types (e.g., vegetables and fruits) on COVID-19 incidences and deaths based on the data from some developed and developing countries that consume the most or least amounts of VF.

### Machine Learning Assessment

Machine Learning (ML) is a branch of Artificial Intelligence (AI) that can be used for predictive analytics [41]. ML provides tools by which large quantities of data can be automatically analyzed. We utilized the established Machine Learning (ML) model in our research to perform a statistical analysis on available on public database in “Kaggle” [42] to examine the effects of different food types on COVID-19 incidences and deaths. Specifically, we used the feature selection method to identify the factors (e.g., diet-related factors) that contribute to the COVID-19 incidences and deaths. In particular, we focus on ML in predictive analysis of vegetables and fruits consumption associated with COVID-19 incidences and deaths. The data generated here is expected in the long run to improve dietary intervention program and efficiencies of health care providers in preventing and treating COVID-19. Below, we introduce the feature selection method.

### Correlation-Based Feature Selection

Correlation-Based Feature Selection (CFS) ranks the feature subset according to the correlation with the class label and other features [43]. Subsets that show high correlation with the class label and less correlation with other features was ranked a higher value. We used the Weka implementation CorrelationAttributeEval to identify top features or factors (e.g., diet-related factors) with the highest rank for COVID-19 incidences (confirmed cases) and COVID-19 deaths. We only executed ML model on the available COVID-19 and diet (vegetable and fruit) data on public repository in “Kaggle”.

## Results

### Assessment of COVID-19 Based on Low Vegetables and/or Fruits Consumption in Developed Countries

Here our results indicate that the majority of developed nations do not eat enough VF. As seen in Table 1, very few people living in most developed countries eat the recommended amount of VF every day, making them more susceptible to COVID-19 infections and chronic diseases such as cancer, diabetes, and heart disease. Data on COVID-19 incidence and death rates are available online at https://www.worldometers.info/coronavirus/countries-where-coronavirus-has-spread/. These data are Updates as of September 19, 2020. The case fatality rate (CFR) was calculated based on the formula below. The case fatality rate (CFR) is the proportion of people diagnosed with a COVID-19 who die from COVID-19 and is, therefore, a measure of severity among detected cases.

$$\text{Case fatality rate }\left[\text{CFR},\;\left(\%\right)\right]=\frac{\text{Number of deaths from disease}}{\text{Number of confirmed cases of disease}}\times 100$$

Table 1 shows the top ten developed countries in the world that were selected for the studies based on the consumption of fewer amounts of vegetables (2.66 to 5.73 kg/person/year) and/or fruits (3.11 to 6.85 kg/person/year). The selected top developed countries eating fewer vegetables and/ or fruits include Australia, Austria, Canada, Denmark, Finland, France, Germany, Hungary, United Kingdom, and the United States of America.

Based on the Case Fatality Rate (CFR) data, the United Kingdom had the highest COVID-19 death rate (10.69%) among the infected people while Austria had the lowest COVID-19 death rate (2.04%) (Table 1). Among the developed nations where people eat the least vegetables and/or fruits, the United States of America had the highest incidence of COVID-19, and hence more affected by the disease. However, the case fatality rate of 2.92% in the general population suggested that the United States of America had the lowest mortality rate when compared to the United Kingdom with 10.69%, France with 7.07%, and Canada with 6.45%. As seen in Figures 1a and 1b, developed countries where people eat low levels of VF have higher COVID-19 infections and deaths.

### Assessment of COVID-19 Based on high Vegetables and/or Fruits Consumption in Wealthy Countries

Here our results indicate that in developed countries where people eat enough/high levels of VF, the COVID-19 death rate is lower even when the incidence of COVID-19 may be high (Table 2). Among countries where there is a high intake of VF, China is the only country with a higher case fatality rate (CFR - 5.43%) from COVID-19. The highest mortality rate in China may due to the fact that it was the first country to be hit by the virus causing the COVID-19, a newly identified pathogen in Wuhan city, Hubei Province, China, on December 12, 2019. The first group of Chinese patients to be affected with COVID-19 was found to be connected with the Huanan South China Seafood Market in the Wuhan city [44]. Table 2 shows the top six wealthy countries in the world consuming the highest amount of vegetables (10.89 to 13.53 kg/person/year) and/or fruits (3.41 to 9.79 kg/person/year). According to our machine learning process, wealthy countries eating the most vegetables and/or fruits include China, Croatia, Korea-South, Kuwait, Malta, Oman, and Turkey.

As shown in Table 2, China has the highest COVID-19 CFR (5.43%) while Malta has the lowest CFR (0.07%). Among the wealthy nations that eat the most vegetables and/or fruits, Turkey has the highest numbers of incident cases and deaths from COVID-19. However, its CFR of 2.47% in the general population indicates that Turkey is the second most affected country after China with a CFR of 5.43%. As seen in Figures 2a and 2b, wealthy countries where people eat the most VF have in general lower COVID-19 incidence and death rates.

### Assessment of COVID-19 Based on high Vegetables and/ or Fruits Consumption in Developing Countries

Here our results indicate that in developing countries where people eat enough, or high levels of VF, there are low COVID-19 incidences. However, case fatality rates of over 5% were observed in two countries including Egypt (5.63%) and Niger (5.93%). Table 3 shows the top seventeen (17) developing countries where people consume the most vegetables (10.07 to 19.29 kg/person/year) and/or fruits (2.28 to 7.92 kg/person/year). These countries include Albania, Algeria, Armenia, Azerbaijan, Bosnia and Herzergovina, Egypt, Guyana, Iran (Islamic Republic), Jordan, Kazakhstan, Kyrgyzstan, Lebanon, Niger, Tajikistan, Tunisia, Uzbekistan, and Vietnam.

Based on the CFR data, Egypt and Niger have the highest COVID-19 death rates rate (5.63% and 5.93% respectively) while Jordan has the lowest rate (0.07%) of COVID-19 death (Table 3). Among the developing countries that eat the most vegetables and/or fruits, Iran (Islamic Republic) has the highest number of SARS-CoV-2 infections and COVID-19 related deaths. However, its case fatality rate of 2.69% is lower than those reported in Niger (5.93%), Egypt (5.63%), and a few other developing countries; indicating that the mortality rate is lower among people who are infected with SARS-CoV-2 in Iran compared to other countries. As seen in Figures 3a and 3b, developing countries where people eat high levels of VF have lower COVID-19 incidence and lower COVID-19 death rates, except a few countries with high death rates.

### Correlation between Vegetables and/or Fruits Intake, COVID-19 Incidences, and Deaths

To assess the relationship between fruits intake and COVID-19 incidences (or deaths) and the relationship between vegetable intake and COVID-19 incidences (or deaths), we calculated the Pearson correlation between fruits intake and COVID-19 incidences (or deaths) and the Pearson correlation between vegetable intake and COVID-19 incidences (or deaths) based on the data obtained from the above-listed developed and developing countries. Table 4 shows the summary of correlation between fruits (or vegetables) intake and COVID-19 incidences (or deaths). In this table, we see that fruits intake is negatively correlated with both COVID-19 incidences and COVID-19 deaths, suggesting fruits intake can help to combat SARS-CoV-2 infection. We also see that vegetable intake is negatively correlated with both COVID-19 incidences and COVID-19 deaths, suggesting vegetable intake can help to combat the SARS-CoV-2 infection.

People eating the recommended amounts of VF are more likely to stay healthy while people eating the least amounts of VF are more likely to be infected with COVID-19 and die from the disease, particularly in developing countries where there is limited environmental sanitation and poor access to medical/health care.

## Results based on the Feature Selection Method

We used correlation-based feature selection (i.e., Weka implementation CorrelationAttributeEval) to identify the factors (e.g., diet-related factors) that contribute to the COVID-19 incidences and deaths. Table 5 presents a list of top nutritional factors with the highest rank of COVID-19 incidences and COVID-19 deaths. In descending order, obesity, eggs, animal products, stimulants, milk-excluding butter, meat, tree-nuts, and animal fats were associated with COVID-19 incidences, while obesity, animal products, eggs, animal fats, milk-excluding butter, meat, tree-nuts, and stimulants were associated with COVID-19 deaths.

## Discussions

Using machine learning process, we identified COVID-19 incidences and associated deaths in different countries, and determined their relationship with vegetables and fruits intakes. The data obtained from machine learning analysis demonstrated that: (a) in developed countries, higher COVID-19 incidence and death rates are found in people who consume lower amounts of vegetables and fruits (VF) compared to people eat high quantities of VF; (b) in developed/wealthy countries where people eat enough or more VF, the incidence and death rates from COVID-19 are lower compared to people from developing countries who also eat a higher amounts of VF; (c) in developing countries where people eat a higher amounts of VF, a lower COVID-19 incidence and death rates are found. However, we observed higher case fatality rates in two countries, particularly in Egypt and Niger. The high mortality rate observed in two developing countries where people eat high amount of VF may due to the lack of preventive and control measures such as hand washing, keeping social distance, wearing masks, poor sanitation, and limited medical interventions.

Healthy eating is well recognized for the maintenance of good health and giving the body the chance of fighting against diseases [18,45,46]. During the course of this work, we found that VF contains novel therapeutic agents for the prevention and treatment of different human diseases including COVID-19. Derivative therapeutic agents from many VF have been reported to inhibit the entry and replication of several coronaviruses. For examples, 3-methylbut-2-enyl)-3’,4,7-trihydroxyflavane (flavonoid) a derivative of *Broussonetia papyrifera* inhibits coronavirus proteases enzyme of MERS_CoV virus [47], 4 -Hydroxychalcone (flavonoid) derivative of *Cinnamomum spp* inhibits viral replication of HCoV-NL63 virus and MERS_CoV virus [48,49]. In addition, natural products such as *Echinacea purpurea* inhibit viral replication of the SARS-CoV virus, MERS-CoV virus, and HCoV-229E virus [50]. *Gentiana scabra* inhibits viral replication and enzymatic activity of SARS-CoV virus [16]. These derivative therapeutic agents from many VF have potential antiviral activities effective against different types of coronaviruses and could be used to prevent and/or treat COVID-19.

Based on the Feature Selection Method data, nutritional factors that contribute to high COVID-19 incidences and COVID-19 deaths include obesity (sugar intake), animal fats, eggs, and stimulants. This year, Zeng and his collaborators investigated 214 COVID-19 patients from three hospitals in Wenzhou, China and compared patients with obesity and metabolic-associated fatty liver disease with non-obese and metabolic-associated fatty liver disease. They found that in patients with obesity and metabolic-associated fatty liver disease was associated with a 6-fold increased risk of severe COVID-19 illness [51]. Furthermore, Kalligeros et al. [52] reported an association between severe obesity and intensive care unit (ICU) using retrospective data obtained from 103 COVID-19 patients hospitalized at three hospitals in Rhode Island, United States.

## Conclusions

The Food and Drug Administration recently approved the emergency use of Pfizer and Moderna COVID-19 vaccine. However, there is a public concern about this new COVID-19 vaccine safety, efficacy, and immunity after the vaccination. In addition, the COVID 19 pandemic has progressed and the scientific community is doing more research to gain a better understanding of different ways to prevent the risk of infection, including Vegetable and Fruit (VF) that could give your body a defense boost. Some medical professionals have used hydroxychloroquine, an anti-malarial drug to treat the infection COVID-19 disease [53,54]. Hydroxychloroquine has also been used in combination with azithromycin to improve the treatment outcomes of COVID-19 patients in clinical practices [53,55]. However, treatment of COVID-19 with hydroxychloroquine alone or combination therapy has resulted in adverse health effects in some patients [56,57]. Therefore, it is urgent to explore alternative drugs from Vegetables and Fruits (VF) for the prevention of COVID-19. We demonstrated that the combined intake of VF prevents and reduces the incidence and mortality rates of COVID-19. In addition to our study, COVID-19 can be prevented by keeping social distancing, hand washing, tracking contact and quarantine, shelter-in-place, environmental hygiene, and wearing face masks. VF are loaded with numerous phytochemicals such as flavonoids, terpenoids, polyphenols, tannins, saponins, alkaloids, and nutrients such as zinc, beta-carotene, vitamins C and E that possess myriad of functions against viral penetration, invasion, replication, expression, assembly, and release. Therefore, high intake of VF may prevent SARS-COV-2, representing a promising chemopreventive agent in the fighting against COVID-19.

Our results are based on geographical locations and the amounts of VF intake in different countries using machine learning process which might be subject to biases if the data collected from different locations are either overestimates or underestimates of actual VF intake. Machine learning based CT analysis has been suggested as a promising screening tool for COVID-19 when compared to viral real-time PCR testing [58]. As seen in the present study with the case of COVID-19, many diseases exhibit large geographical variations which are often not explained despite abundant research [59]. Therefore, further studies including preclinical and clinical trials are needed to confirm the health benefits of VF to COVID-19 patients.

## Figures and Tables

**Figure 1a:**
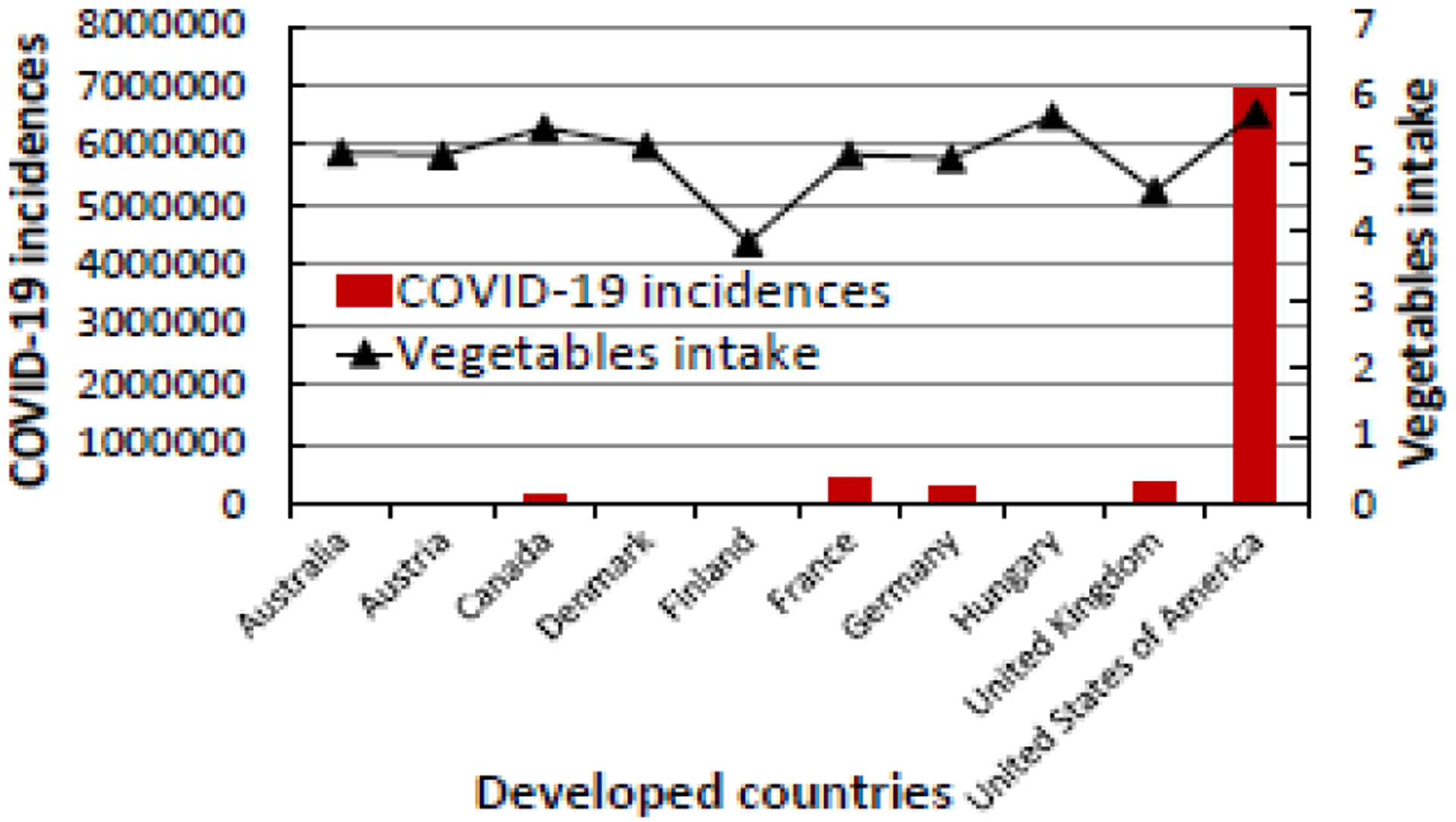
The relationship between COVID-19 incidences and vegetable intake in the top ten developed countries in the world that eat fewer amount of vegetables (2.66 to 5.73 kg/person/year).

**Figure 1b:**
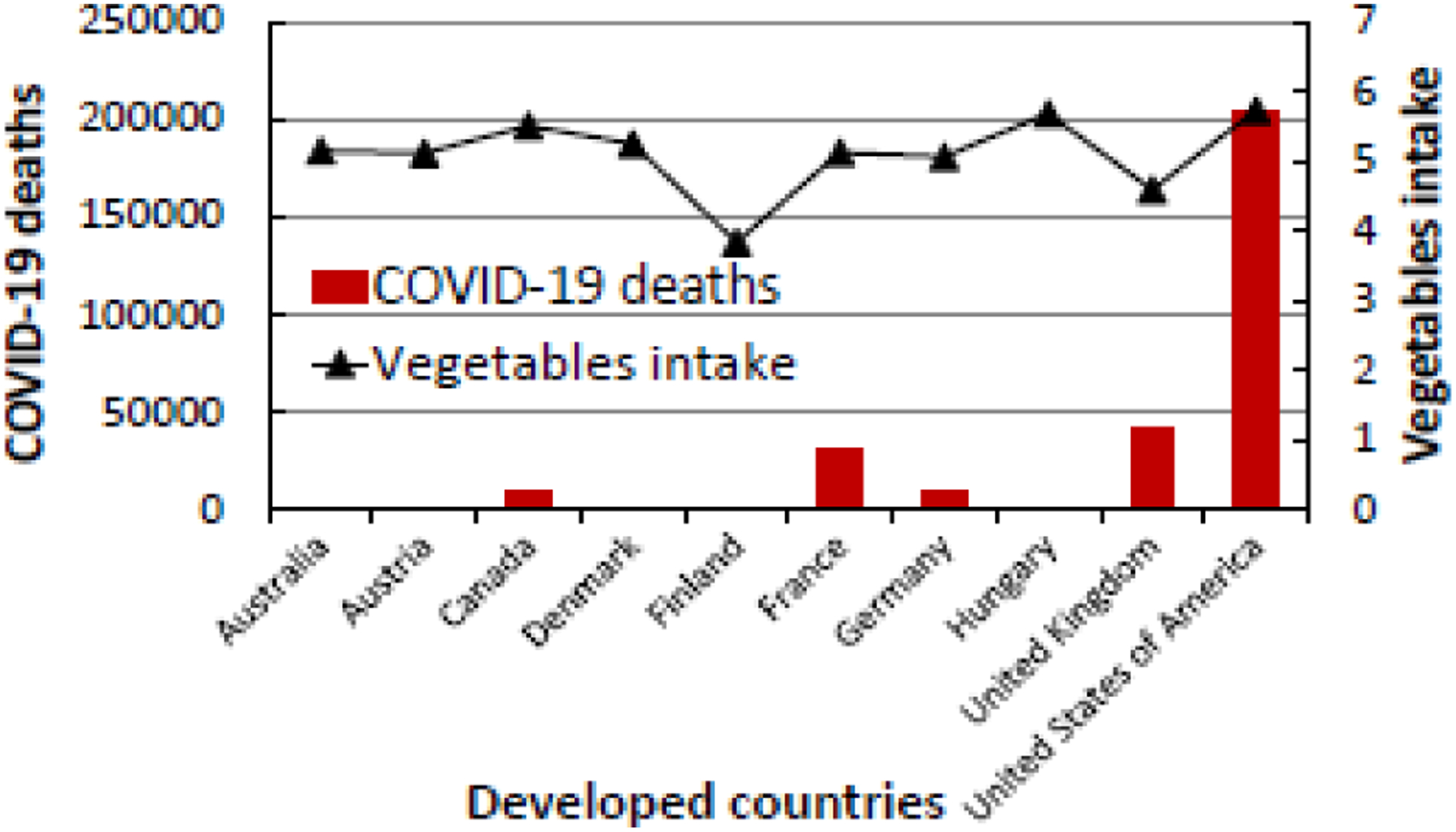
The relationship between COVID-19 deaths and vegetable intake in the top ten developed countries in the world that eat fewer amount of vegetables (2.66 to 5.73 kg/person/year).

**Figure 2a:**
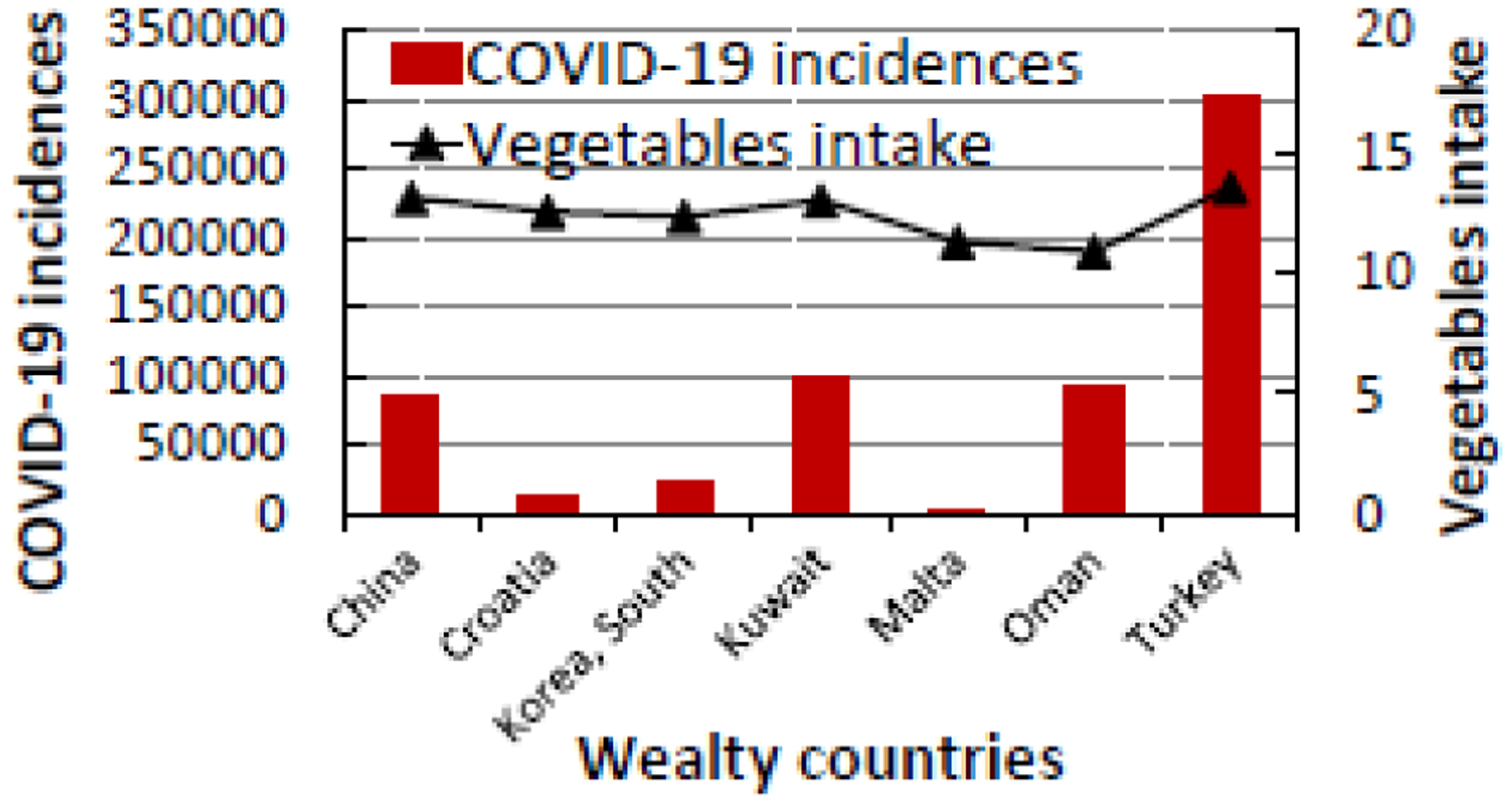
The relationship between COVID-19 incidences and vegetable intake in the top six developed countries in the world that consume the most amount of vegetable (10.89 to 13.53 kg/person/year).

**Figure 2b:**
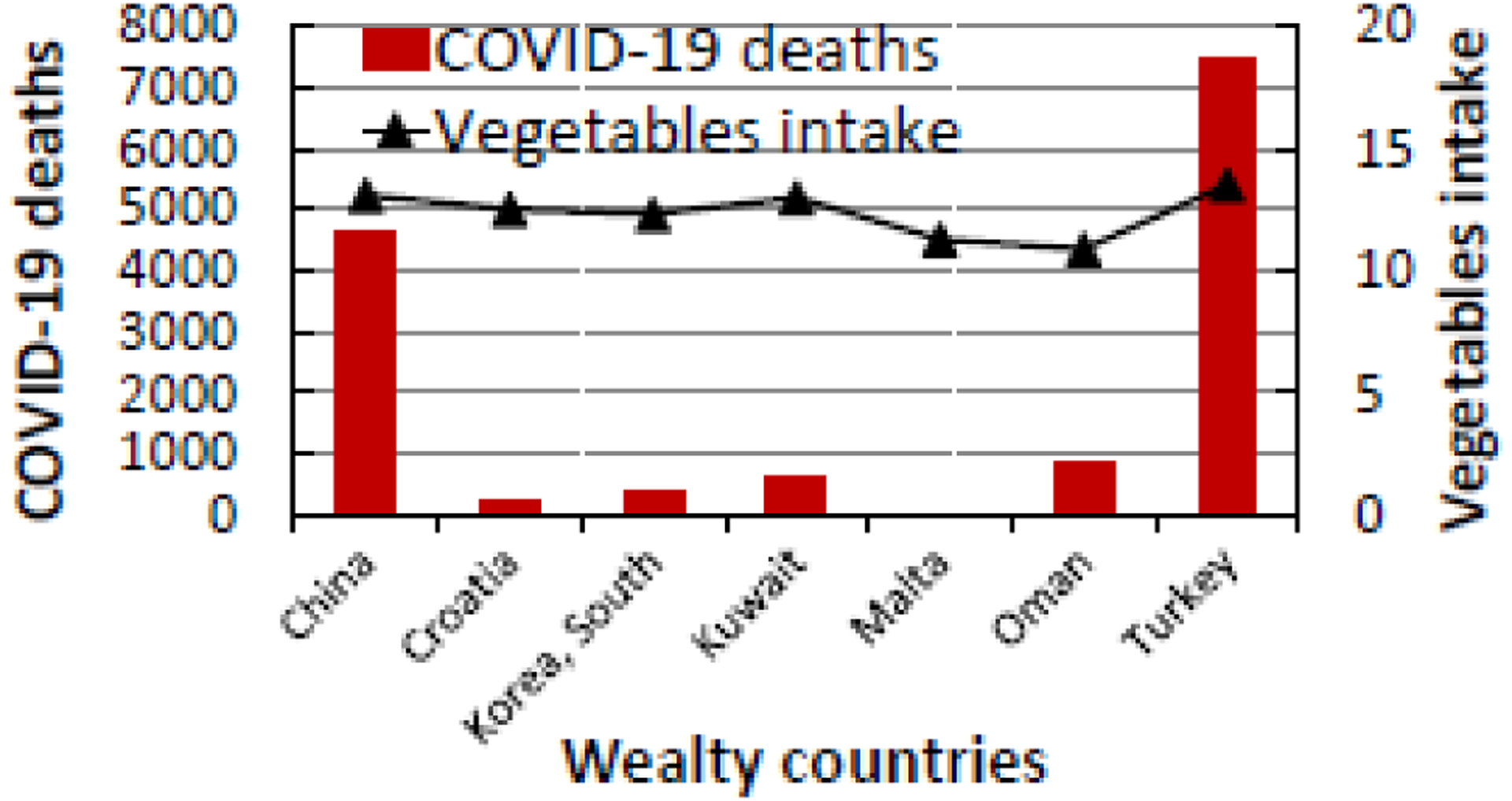
The relationship between COVID-19 deaths and vegetable intake in the top six developed countries in the world that consume the most amount of vegetable (10.89 to 13.53 kg/person/year).

**Figure 3a:**
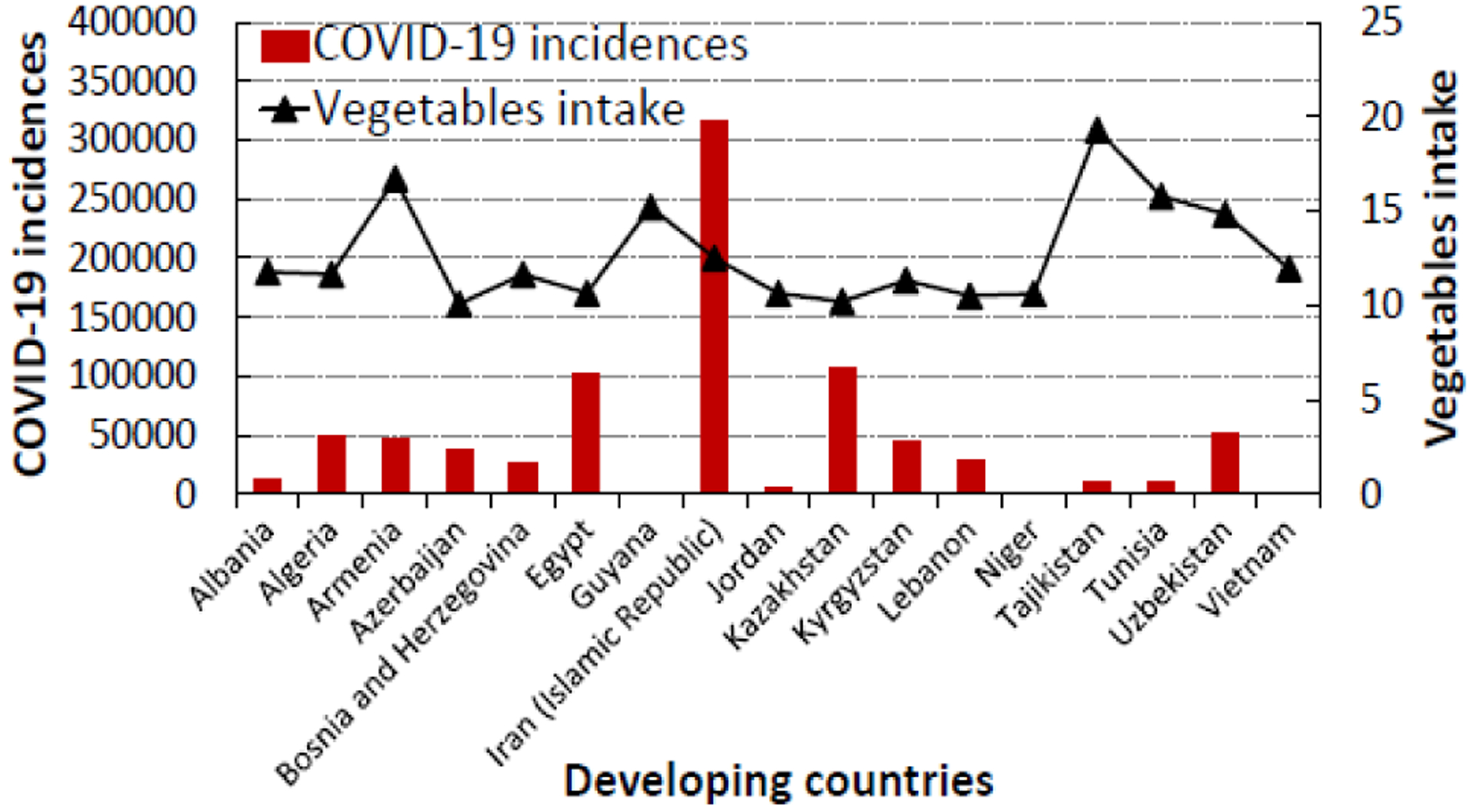
The relationship between COVID-19 incidences and vegetable intake in the top seventeen (17) developing countries in the world that consume the most vegetables (10.07 to 19.29 kg/person/year).

**Figure 3b:**
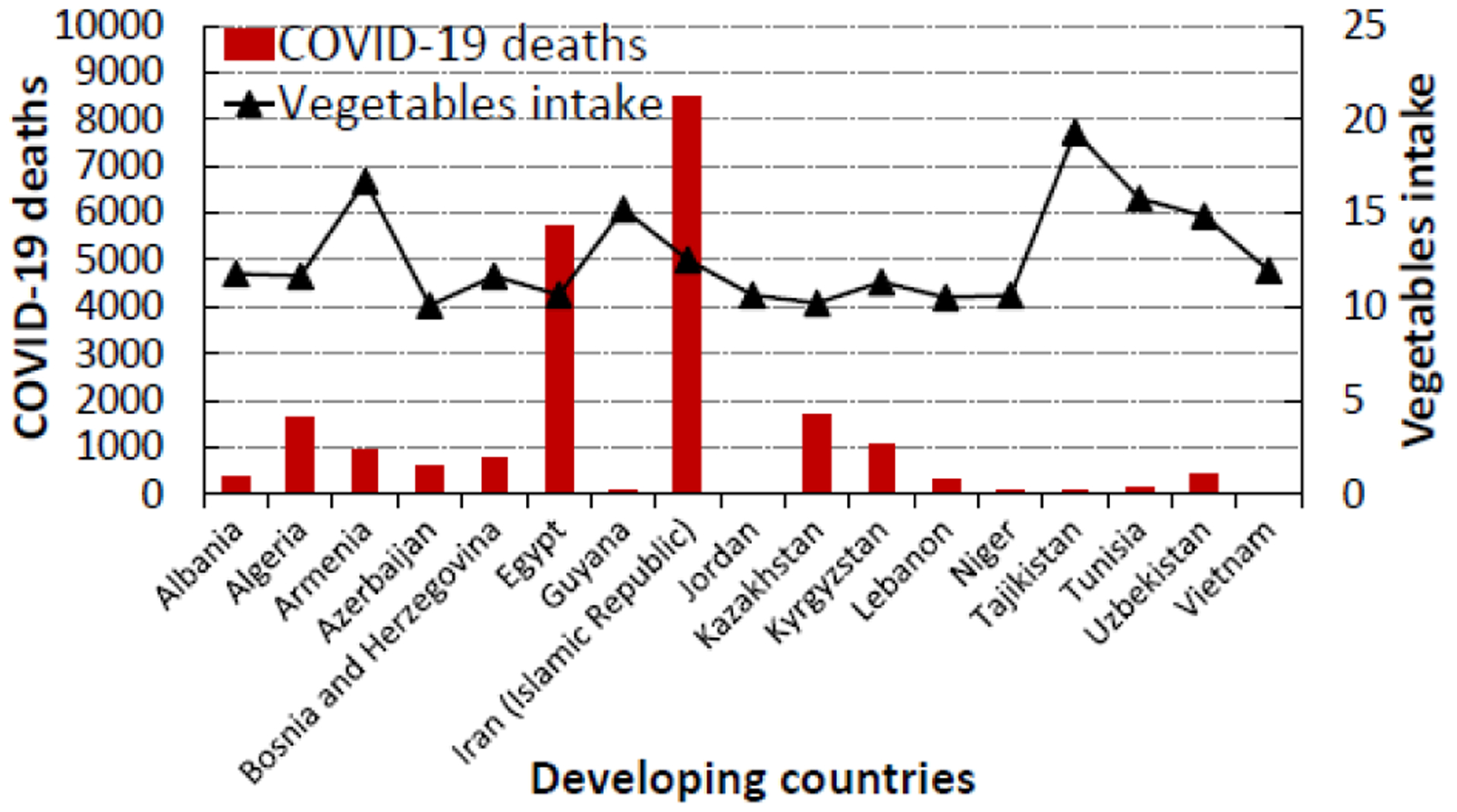
The relationship between COVID-19 deaths and vegetable intake in the top seventeen developing (17) countries in the world that consume the most vegetables (10.07 to 19.29 kg/person/year).

**Table 1:** Top ten developed countries in the world consuming the lowest amount of vegetables and fruits.

DEVELOPING COUNTRIES	VEGETABLES INTAKE	FRUITS INTAKE	COVID-19 INCIDENCES	COVID-19 DEATHS	CASE FATALITY
Australia	5.1406	4.1883	26,885	844	3.14%
Austria	5.1098	4.6069	37,474	765	2.04%
Canada	5.4961	5.0692	142,745	9,211	6.45%
Denmark	5.2431	3.1098	142745	635	2.83%
Finland	3.8325	3.1864	22,436	339	3.80%
France	5.1223	4.878	442,194	31,274	7.07%
Germany	5.0542	3.6131	271,840	9,466	3.48%
Hungary	5.6788	3.0852	16,920	675	3.98%
United Kingdom	4.5851	4.9551	390,358	41,759	10,69%
United States of America	5.7249	4.5432	6,955,007	203,565	2.92%

**Table 2:** Top six wealthy countries consuming the highest amount of vegetables and fruits.

WEALTY COUNTRIES	VEGETABLES INTAKE	FRUITS INTAKE	COVID-19 INCIDENCES	COVID-19 DEATHS	CASE FATALITY
China	13.099	4.2451	85,269	4,634	5.43%
Croatia	12.5188	3.4094	14,725	244	1.66%
Korea, South	12.3349	3.6993	22,893	378	1.65%
Kuwait	12.9953	5.3058	99,049	581	0.59%
Malta	11.2856	4.631	2,699	19	0.07%
Oman	10.8902	9.787	91,753	818	0.09%
Turkey	13.5284	6.8321	301,348	7,445	2.47%

**Table 3:** Top seventeen developing countries where people consume the most vegetables and/or fruits.

DEVELOPING COUNTRIES	VEGETABLES INTAKE	FRUITS IN TAKE	COVID-19 INCIDENCES	COVID-19 DEATHS	CASE FATALITY
Albania	11.7753	6.7861	12,226	358	2.92%
Algeria	11.6484	6.3801	49,623	1,665	3.35%
Armenia	16.7019	6.0989	47,154	928	1.97%
Azerbaijan	10.0755	4.7988	39,042	574	1.47%
Bosnia and Herzegovina	11.6394	4.6409	25,217	758	3.00%
Egypt	10.6559	6.9387	101,772	5,733	5.63%
Guyana	15.2061	5.7954	2,102	62	2.95%
Iran (Islamic Republic)	12.5207	7.9235	315,597	_8,491_	2.69%
Jordan	10.6308	4.6624	4,540	_30_	0.07%
Kazakhstan	10.2055	3.2145	107,199	_1,671_	1.56%
Kyrgyzstan	11.3219	2.2846	45,335	_1,063_	3.76%
Lebanon	10.5128	6.9852	28,297	_286_	2.42%
Niger	10.6117	2.5503	1,183	_69_	5.93%
Tajikistan	19.2995	3.7863	9,303	_73_	0.08%
Tunisia	15.7731	5.4307	9,110	_138_	1.51%
Uzbekistan	14.8354	5.215	50,872	_425_	0.08%
Vietnam	11.9508	5.9029	1,068	_35_	3.28%

**Table 4:** Summary of correlation between Fruits (or Vegetables) Intake and COVID-19 Incidences (or Deaths).

	Fruits Intake	Vegetables Intake
COVID-19 Incidences	−0.0282	−0.2141
COVID-19 Deaths	−0.0349	−0.2736
P-values	0.6349	0.0353

**Table 5:** Summary of top factors with the highest rank of COVID-19 incidences and COVID-19 deaths based on Correlation Attribute Eva.

COVID-19 Incidences (Confirmed Cases)	COVID-19 Deaths
Obesity	Obesity
Eggs	Animal Products
Animal Products	Eggs
Stimulants	Animal fats
Milk - Excluding Butter	Milk - Excluding Butter
Meat	Meat
Tree-nuts	Tree-nuts
Animal fats	Stimulants
